# Regulation of Embryonic Wound Healing by Matrix Metalloproteinases in *Xenopus laevis* Tailbud Stage

**DOI:** 10.1111/wrr.70134

**Published:** 2026-02-18

**Authors:** Daniel Kraus, Paulina Kikinderova, Pavel Abaffy, Dominika Kadlcikova, Ravindra Naraine, Radek Sindelka

**Affiliations:** ^1^ Laboratory of Gene Expression Institute of Biotechnology of the Czech Academy of Sciences – BIOCEV Czech Republic

**Keywords:** actin‐myosin ring, adult and embryonic healing, AP‐1 pathway, *mmp*, *Xenopus laevis*

## Abstract

Scarless wound healing remains a key goal in regenerative research. However, the gene regulatory networks and mechanisms behind this process are still not fully understood, despite the use of high‐throughput analyses and various research models. In our study, we focus on the intermediate phase and examine the role of remodelling genes called matrix metalloproteinases (MMPs) in the *Xenopus laevis* model. Through temporal bulk RNA‐Seq analysis, we identified the *fos/jun* (AP‐1) combination expressed during the early phase and four main *mmps* (1, 7, 8, and 9) expressed during the intermediate phase of healing. Using specific MMP inhibitors and morpholino‐oligonucleotides targeting *fos/jun*, we found that *mmp* expression depends on stress response and that they are crucial for embryonic wound healing. We also analysed other published healing data sets (single cell and bulk RNA‐Seq) and observed a strong correlation of our observations with healing in mammals. In summary, our study indicates that wound healing is a conserved biological process that begins with a stress response involving AP‐1. It then progresses through the stage of extensive healing activity, including the expression induction of specific *mmp* genes during the remodelling phase.

## 1 Introduction

Healing processes and their regulation are important areas of study in medical and biological research because they have great potential to improve treatment methods. In this context, healing is defined as the process of restoring skin integrity and homeostasis, which starts immediately after injury [1]. Wound healing is a highly controlled process. We can distinguish two main types: adult and embryonic healing [2]. Understanding these processes is crucial for advancing scientific knowledge and development of new treatments.

Adult wound healing is a complex process that typically takes longer and is generally better understood than embryonic healing. It ends with the formation of a scar, which often has functional and aesthetic limitations. This type of healing consists of four major phases: haemostasis, inflammation, proliferation and remodelling [3]. The first stage, haemostasis, occurs immediately after injury and involves blood vessel constriction along with fibrin clot formation to stop bleeding. During this phase, platelets are important because of their ability to release growth factors that help recruit neutrophils and monocytes [4, 5, 6]. The next stage, inflammation, is characterised by the activity of neutrophils, macrophages, and other immune cells that work together to clear debris and fight infections [2, 3, 4, 5, 6, 7]. During this stage, epithelial cells and fibroblasts become activated [6]. The third stage, proliferation, is characterised by active fibroblasts that promote tissue regeneration. This stage involves the buildup of extracellular matrix components, including fibronectin, proteoglycans and collagen, which replace the initial fibrin matrix. After angiogenesis and epithelialisation, granulation tissue matures during the final stage (remodelling). This last phase results in a scar rich in collagen, which has less strength and functionality compared to uninjured tissue [8].

In contrast to adult wound healing, embryonic wound healing is less complex, faster and scarless [9]. Still, our knowledge about the signalling molecules and regulatory mechanisms involved remains limited. The embryonic healing process that is studied in *Xenopus* embryos has three phases: early, intermediate and late [10]. The early phase starts immediately after the injury is initiated. It involves several factors such as calcium, nitric oxide (NO) and reactive oxygen species (ROS). These are also crucial for actin‐myosin ring formation as well as resisting infection, cytoprotection and initiating wound healing. The assembly of the actin‐myosin ring at the wound edge assists with wound closure through cell‐specific contractile movement [3, 11]. Healing‐specific gene expression triggers the second, intermediate phase. During this phase, primitive myeloid cells migrate to the healing site and depend on the activity of remodelling enzymes called matrix metalloproteinases (MMPs). The late phase lasts several more hours and involves further remodelling, cell proliferation and differentiation.

Both adult and embryonic healing processes rely on the enzymatic activity of MMPs. Although the functions of these enzymes are well studied in adults, they remain unclear in embryos. Due to several duplication and fusion events in the vertebrates, there are numerous *mmp* members, which makes it difficult to determine their individual functions (there are 24 *mmps* in *Homo sapiens* , 23 in *Mus musculus* and 20 in *Xenopus laevis* ) [12, 13]. MMPs/*mmps* are regulated not only at the level of enzymatic activity but also at the level of their expression [14]. Embryonic expression of *mmps* is generally low, but their expression increases after injury. In contrast to the relatively late activation of *mmps*, several signalling pathways such as AP‐1, MAPK, STAT, the Smad family and NF‐κB are activated during early phases, and one of their roles is to stimulate *mmp* expression [15]. This relationship is encoded in the regulatory sequences of *mmps* through regulatory elements [16].

A direct relationship between MMPs and AP‐1 in wound healing has already been proposed [17]. AP‐1 is a transcription factor that regulates gene expression in response to various stimuli [18] and controls several cellular processes, including differentiation, proliferation and apoptosis [19]. Jun proteins, which are part of the AP‐1 complex, can form both homo‐ and heterodimers, while Fos proteins only form heterodimers [20, 21]. The Jun/Fos dimer is more stable and has greater transcriptional activity than other combinations [22]. The dimerisation partners of Jun affect its role in gene activation and cell cycle regulation [23].

In this study, we used various tools to reveal the role of *mmps* in connection with AP‐1 during wound healing in *Xenopus laevis* embryos. We found that *mmp1.S, mmp7.L, mmp8.L* and *mmp9.S* are predominant and necessary for the healing process and play a role in the actin ring formation and ECM remodelling. We used our previously published single cell RNA‐seq and other available mouse and human healing datasets to validate whether these genes are primarily expressed and how they are involved in the wound healing.

## 2 Materials and Methods

### 2.1 Preparing of Embryos

Adult *Xenopus laevis* females were injected with 400 units of HCG hormone to stimulate egg production for the following day. Fertilisation was performed in vitro, and fertilised embryos were cultured in 0.1×MBS solution (Modified Barth's Saline: 8.8 mM NaCl, 0.1 mM KCl, 0.5 mM HEPES pH 7.8, 0.07 mM CaCl_2_, 0.1 mM MgSO_4_, 0.25 mM NaHCO_3_) at 15°C.

### 2.2 Inhibition of MMPs and AP‐1

We used four chemical inhibitors to target various MMPs with the goal of assessing their role during early healing stages. For each inhibitor, embryos at stage 26 were incubated in the culture medium with the inhibitor at a given concentration for 1 h before injury. A scratch injury measuring 110–130 μm was made on the lateral side of the embryos using fine forceps. The embryos were then incubated in the inhibitory medium at 15°C and fixed at specific time points (1 and 3 h after injury) for immunohistochemistry and RT‐qPCR. Twenty embryos per condition in at least two independent experiments were used in all inhibitory assays.

The first chemical inhibitor D609 (Calbiochem, 251,400) is a selective inhibitor of phosphatidylcholine (PC)‐specific phospholipase C and a broadly expected general MMP inhibitor. It was used at a concentration of 0.02 mM within the culture medium. The second chemical inhibitor, (MMP‐8 Inhibitor I) (Cayman Chemical, 21,852), targets specifically MMP8 activity. It was used at a concentration of 4 μM within the culture medium. The third inhibitor GM6001 (Sigma Aldrich, CC10) was added to the medium at 100 μM. GM6001 is an inhibitor that is a broad‐spectrum MMP inhibitor. It targets the zinc‐binding site in MMP genes. It also effectively blocks MMP activity and it was reported to inhibit MMP 1, 2, 7, 8, 9, 12, 13, 14, 16, 26. The fourth inhibitor Batimastat (Abcam, ab142087) was added to the medium at concentrations ranging from 10 μM to 100 μM. and it is also a broad‐range MMP inhibitor. It inhibits mainly MMP 1, 2, 7, 9 by chelating the zinc ion in the active site of the enzyme.

In addition to the chemical inhibitors, we also utilised morpholino oligonucleotides (MO) to directly target AP1 genes. Morpholino oligonucleotides (MO) were designed and purchased from Gene Tools LLC (Philomath, OR, USA). We used four MOs against *fos* and *jun* to target both expression variants (L/S). Sequences were:

*fos.S* (TTCGGATAATAGACTTACCTGTGGA),

*fos.L* (AGGTCAGATAATTACCTGCTCCATT),

*jun.S* (GTAGTTTCCATCTTTGCGTTCATAC),

*jun.L* (GCTTATGTCAGTGTGACGACACCAA).

Fertilised embryos at the one cell stage were transferred into 0.1× MBS with 3% Ficoll and microinjected with the MO (17 ng) into the animal pole before the first cell division. Embryos were incubated at 15°C (each contained at least 20 embryos per condition). We prepared equimolar mixtures of *fos.S* and *fos.L* MOs or *jun.S* and *jun.L* MOs. We tested their inhibitory effect using an injection of either *fos.S/L* or *jun.S*/*L* MOs or a combination of *fos*: *jun*. We found that a mixture of MOs had a stronger effect than individual MOs, and the most effective was a combination of *fos: jun* MOs 3:1 in comparison with 1:1 or 2:1, all at the final amount of 17 ng per embryo.

Pictures of the injury were taken with a binocular light microscope and a Nikon camera at specific time points (0, 1, 3 h after injury) to track its closure. The wound's closing speed was analysed in ImageJ (NIH). The wound area was measured and normalised to time zero. Wound‐healing closure data for both chemical inhibitor‐treated and morpholino oligomer‐treated samples were analysed in R (version 4.4.2). A two‐way ANOVA test was used with treatment (chemical inhibitors or morpholino oligomers) and time points as factors, followed by Dunnet's post hoc test to compare each treatment group with the corresponding control at each time point (*p* < 0.05 *, *p* < 0.01 ** and *p* < 0.001 ***).

### 2.3 Immunofluorescence Staining

Immunohistochemistry was performed according to Sive et al. [24] Actin was stained using Alexa Fluor 488–conjugated phalloidin (1:1000; Life Technologies, A12379). Laminin was detected using a primary anti‐Laminin antibody (1:150; Sigma‐Aldrich, L9393) and a secondary goat anti‐rabbit IgG antibody conjugated to Alexa Fluor 488 (1:500; Life Technologies, A11008). Images were obtained on the Zeiss confocal microscope Carl Zeiss LSM 880 NLO (Carl Zeiss AG) and analysed in ZEN (blue edition) software (Carl Zeiss AG). 10 embryos per condition in two independent experiments were collected and analysed.

### 2.4 RT‐qPCR

Two experiments were performed. In the first, we measured the levels of *mmps* only in the wound area by dissecting the healing tissue surrounding the injury at various time points (stage 26). In the second experiment, we determined the levels of *mmps* in whole embryos to assess injury‐associated changes or to identify *mmps* whose levels remained unchanged (stage 40). Embryos were collected and dissected during wound healing, and samples were stored at −80°C. Following the standard protocol, total RNA was extracted using TRI Reagent (Sigma‐Aldrich, T9424). cDNA was synthesised from 100 ng of total RNA using MAXIMA H Minus Reverse Transcriptase (Thermo Scientific, EP0751). RT temperature conditions were: 25°C for 10 min, 50°C for 30 min and 85°C for 5 min. qPCR was performed using iQ SYBR Green Supermix (Bio‐Rad, 1,708,880) and C1000 Thermal Cycler CFX384 Real‐time system (Bio‐Rad) with thermocycling conditions: denaturation for 95°C for 3 min, followed by 50 cycles of 95°C for 15 s, 60°C for 20 s and 72°C for 20 s. Melting curve analysis was performed in all experiments to control for PCR specificity. Ten embryos per condition in two independent experiments were collected and analysed.

Gene expression changes during healing at stage 40 and stage 26 were analysed in R (version 4.4.2). Differences between each time point for each specific *mmp* genes were assessed using one‐way ANOVA followed by Tukey's multiple comparison test (for stage 40) or Dunnet's test (for stage 26) (*p* < 0.05 *, *p* < 0.01 ** and *p* < 0.001 ***).

### 2.5 Bulk RNA‐Seq

RNA‐Seq data regarding the temporal aspects of embryonic wound healing in *X. laevis* has already been described by Abaffy et al. [10] and was used for this research. In brief, wounded tissue samples from embryos at stage 26 were collected at 0, 0.5, 1.5, 3, 6 h after the injury. RNA was isolated using TRI Reagent (Sigma‐Aldrich, T9424) following the standard protocol. Libraries were prepared from 200 ng of total RNA using SureSelect Strand‐Specific RNA Library Prep for Illumina Multiplexed Sequencing (Agilent, G9691) according to the manufacturer's protocol. Final libraries were equimolar pooled and sequenced on NextSeq 500 using 2 × 75bp HighOutput mode. The data are available at NCBI's Gene Expression Omnibus under GEO Series accession number GSE116667. The pre‐processed data were extracted and the expression levels of *mmp1.S, mmp7.L, mmp8.L* and *mmp9.S* were further analysed.

Human microarray expression data from Leonardo et al., 2023 [25] was downloaded from GEO (accession number GSE209609). In this study, authors used human palate and skin tissues to uncover gene expression during the first 7 days after the injury. Mouse microarray expression data from Chen et al., 2010 [26] was also downloaded from GEO (accession number GSE23006). In this study, authors focused on comparing the transcriptomes of oral mucosal and skin wound to find the differences between these two sites. The downloaded data was checked for previous normalisation by visually inspecting the expression data distribution per sample as a boxplot. The z‐scores of the normalised gene expression were calculated for each gene of interest across the time points and heatmaps of these data were plotted.

### 2.6 Single Cell RNA‐Seq

Single cell RNA‐Seq data following the temporal aspects of embryonic healing and regeneration in *X. laevis* has already been done and described by Sindelka et al., 2024 [27]. In brief, single cell experiment was performed using 0, 1, 3, 12 h post injury of *X. leavis* tadpole tails at stage 42. We expect that early phase of regeneration following tail amputation reflects healing. Healing tissues from 50 embryos were collected per sample. Then the tissue was dissociated into cell suspensions. Sequencing libraries were prepared according to the manufacturer's manual Chromium Single Cell 3′ Reagent Kits User Guide (v 3.1)” and 2400 cells per sample were loaded into Chromium chip. The sample libraries were then pooled and sequenced on Illumina NovaSeq 2000, targeting 100,000 read pairs per cell. The data is available at NCBI's Gene Expression Omnibus under GEO Series accession number GSE245312. For this study the previously pre‐processed and normalised data were used. The pathway activity scoring was performed to visualise pathways activity in cell types. The score is the average expression of a set of genes after subtracting the average expression of a reference set of genes. Score analysis was calculated using scanpy package 1.10.4 (Python 3.11) with its build‐in functions. Expression of *mmp9*, *itgb1*, *pxn, vcl* and *actn1* were used for the score of migration. Expression of *fos, jun, junb, atf3* were used for AP1 pathway score and remodelling phase (*mmp1, mmp2, mmp3, mmp7, mmp8, mmp9, mmp10, mmp13, mmp14*) were used for wound healing. Results were visualised in matplotlib 3.10.0 package. Similarly, the Figure S1 was done to show expression of individual *mmp* genes in different cell types in different time points. The package scanpy 1.10.4 in Python 3.11 was used to calculate and visualise the results with its build‐in functions. Expression of individual *mmp* genes was calculated in each cell types and each time points and visualised with heatmap.

Mouse single cell RNA‐seq data from Guerrero‐Juarez et al., 2019 [28] (GEO accession number GSE113854) and human single cell RNA‐seq data from Liu et al., 2025 [29] (GEO accession number GSE241132) were downloaded. The data were processed using Seurat package (5.2.1) in R (4.4.2). For mouse single cell RNA‐seq [28], cells that displayed < 8000 UMI/cell and < 2500 genes/cell, and no more than 8% mitochondrial gene expression were kept. Data were then log normalised and scaled followed by Principal Component Analysis (PCA). Then clusters of cells were identified with the use of top 40 PCs and then the dimensionality reduction was performed (t‐SNE). Annotation of the clusters were done using the author's marker genes. The average expression of the genes of interest for each cell cluster was plotted using a dot plot. For the human single‐cell RNA‐seq [29], the author's already preprocessed data was used, including their determined cell clusters and annotations. The data were log normalised and scaled. The average expression of the genes of interest for each cell cluster was plotted using a dot plot.

### 2.7 In Situ Hybridisation

Whole‐mount in situ hybridisation was used to determine what cells express *mmp1, mmp7, mmp8* and *mmp9*. It was performed according to the protocol of Sive et al. [24] Embryos per each condition (10 embryos for each condition) were fixed in (paraformaldehyde) PFA overnight, dehydrated in methanol and stored in a −20°C freezer. The proteinase K treatment was not used. Samples were imaged on a stereomicroscope (Nikon SMZ 1500) and processed using Zoner Photo Studio 17.

## 3 Results

### 3.1 High Expression of Mmps During Intermediate Phase of Healing

**FIGURE 1:**
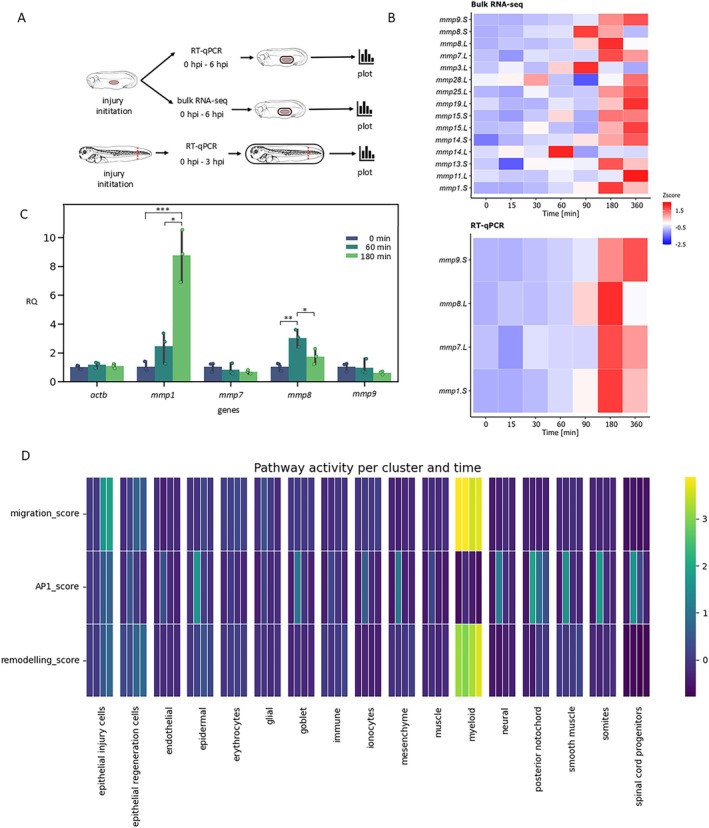
*mmps* are involved in embryonic wound healing. (A) Scheme of experimental procedure. (B) Bulk RNA‐seq data show the expression profiles of *mmp*s important in wound healing and RT‐qPCR validation show the expression profiles of four selected *mmp*s (the graphs show z score of normalised gene expression). (C) Expression of selected *mmps* in the whole embryos during healing studied in embryos of stage 40 (*n* = 3) RQ—relative quantity normalised to time 0 (hpi—hours post injury), one‐way ANOVA followed by Tukey's multiple comparison test, *p* < 0.05 *, *p* < 0.01 **, *p* < 0.001 ***. (D) Single cell RNA‐seq data show which pathways are active across cell types in different time points in order 0, 1, 3, 12 h.

Our analysis of the previously conducted bulk RNA‐seq experiment during *Xenopus* embryonic wound healing (Figure 1A) showed that numerous *mmps* were expressed in the phase we called intermediate (Figure 1B). At the wound site, *mmp1.S, mmp7.L* and *mmp8.L* showed a clear pattern with a peak around 3 h post‐injury, while *mmp9.S* reached its highest expression at 6 h after the injury (Figure 1B). We confirmed these bulk RNA‐Seq results with independent RT‐qPCR and observed the same expression profiles (Figure 1B).

We continued with determining whether the increased *mmp* expression (*mmp1.S*, *mmp7.L*, *mmp8.L*, *mmp9.S*) at the injury site was potentially caused by a global increase in expression throughout the entire embryo or if it resulted from cell‐specific migration to the injury site, leading to a localised expression change (increased at the wound site but unchanged in the whole embryo). To determine this, we performed RT‐qPCR analysis of the observed *mmps* in the whole embryo (stage 40, time points: 0, 1 and 3 h after the injury) and normalised their levels to those in control (uninjured) embryos. We observed that *mmp1* and *mmp8* showed an increased expression in the entire embryo, suggesting injury‐associated upregulation, while *mmp7 and mmp9* maintained the same levels per embryo (Figure 1C).

Additionally, we analysed our *Xenopus* single‐cell RNA‐seq data for pathway activity across different cell types (Figure 1D). The results suggested cell roles during wound healing. We can see that epidermal cells that express *mmp1.S* and *mmp8.L* genes are not highly active during migration, and in addition, their expression level is minimal at time 0, suggesting expression activation following injury. We can observe that these cells show an injury‐associated increase in abundance and are enriched at the wound site during wound healing. In contrast, *mmp7.L* and *mmp9.S* are highly expressed genes primarily in the myeloid cells. Previous literature suggested their active migration to the injury site [30, 31, 32] and our scoring parameters also show increased migration and remodelling features. Similar results are shown in the Figure S1. This figure shows the expression of *mmp1.S, mmp7.L, mmp8.L*, and *mmp9.S* in different cell types across different time points. Our results suggest that *mmp1.S* and *mmp8.L* display localised expression in injury‐related epidermal cells, and *mmp7.L* and *mmp9.S* are mainly expressed in migratory myeloid cells.

### 3.2 Mmps Are Necessary for Embryonic Wound Healing

**FIGURE 2:**
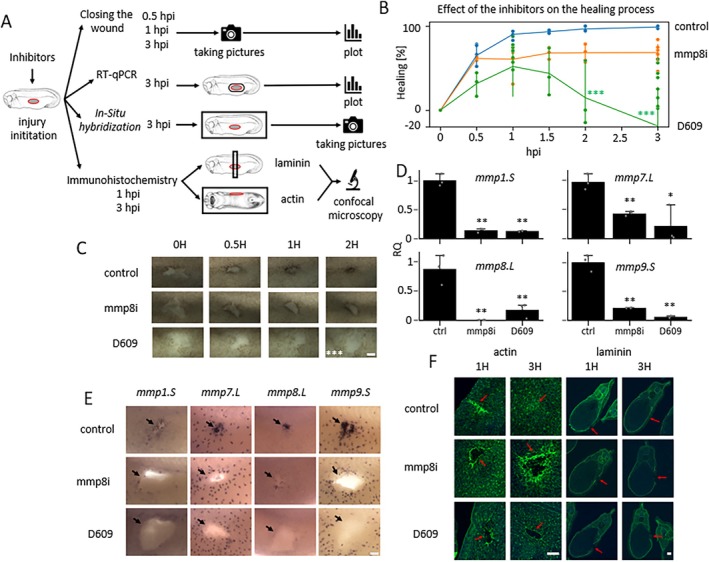
The effect of the MMP inhibition during wound healing. (A) Scheme of the experimental procedure. (B) Quantification of wound closure in time (x axis) calculated as percentage normalised to time 0—Injury (y axis), *n* = 3–13 per condition, two‐way ANOVA followed by Dunnet's post hoc test, *p* < 0.05 *, *p* < 0.01 **, *p* < 0.001 ***. (C) Brightfield images of wound assays from the representative embryos (scale bar 100 μm). (D) Expression of *mmps* in wound site studied using RT‐qPCR in 3 h after the injury (control *n* = 3, mmp8i *n* = 3, D609 *n* = 2), one‐way ANOVA followed by Dunnet's post hoc test, *p* < 0.05 *, *p* < 0.01 **, p < 0.001 ***. (E) Visualisation of selected *mmps* expression by in situ hybridisation in representative embryos in 3 h after the injury (*n* = 10, scale bar 50 μm). (F) Actin ring formation defect (phalloidin staining) and problem of basal lamina formation showed with red arrow (Laminin staining) in 3 h after the injury (*n* = 10, scale bar 100 μm). (mmp8i—Inhibitor of *mmp8*).

The *mmps* expression peak at 3–6 h post‐injury suggests their relevance during intermediate and late phases of healing. We performed transient MMPs inhibitory experiments to study their roles using either the global MMP inhibitor D609 or the MMP8 specific inhibitor (mmp8i) (Figure 2). Both inhibitors led to a weaker wound closure relative to the control (Figure 2B,C). Control embryos closed 97.3% of their wounds in 3 h, in contrast to the embryos treated with D609 inhibitor (18.8%) and the embryos with *mmp8* inhibitor (68.6%) (Figure 2B).

We also determined the expression levels of other *mmps* in the wound area following inhibition. We detected a significantly decreased expression (Figure 2D). The same decrease of *mmps* was also observed when using in situ hybridisation. Application of the MMP8 inhibitor led to low expressions of *mmp1.S* and *mmp8.L* around the wound and a migratory defect of the myeloid cells, as shown using *mmp7.L* and *mmp9.S* staining (Figure 2E). The same pattern was observed after application of the D609 inhibitor (Figure 2E).

Actin‐myosin ring is usually formed around the edges of the wound and helps the wound to close by constricting along the opposite sites. Its formation is crucial for successful healing initiation. We performed immunohistochemistry of actin to visualise its formation. Control embryos showed a clear actin‐myosin ring formed that pulled together at the late healing phase. In contrast, inhibited embryos showed poor actin‐myosin ring formation for both inhibitors and the absence of wound closure after 3 h (Figure 2F). Remodelling of the extracellular matrix (ECM) is also an essential step for healing, and it depends on MMP activity. We performed Laminin immunohistochemistry to visualise basal lamina remodelling following injury. The Laminin layer was reformed at the wound site in control embryos versus no reformation in both the inhibited embryos (Figure 2F).

**FIGURE 3:**
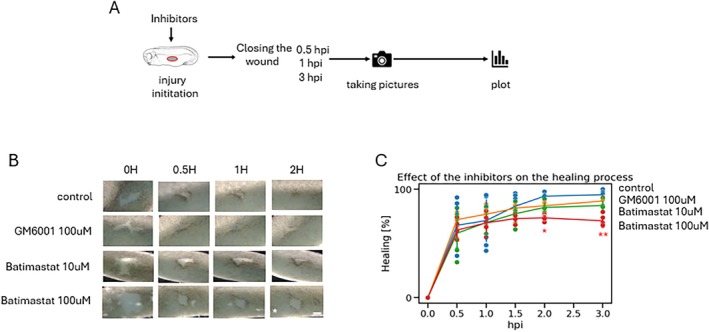
Inhibition of wound healing with broad range inhibitors. (A) Scheme of the experimental procedure. (B) Brightfield images of wound assays from the representative embryos (*n* = 5, scale bar 100 μm). (C) Quantification of wound closure in time (x axis) calculated as percentage normalised to time 0—injury (y axis), *n* = 5 per condition, two‐way ANOVA followed by Dunnet's post hoc test, *p* < 0.05 *, *p* < 0.01 **, *p* < 0.001 ***.

To validate specificity of *mmps* for healing, we used two others widely used *mmp* inhibitors GM6001 and Batimastat. Both inhibitors showed reduced wound closure, especially during the intermediate healing phase (Figure 3B,C). Wound closure for GM6001 remained closed for about 89.2% after 3 h post injury and for Batimastat 84.9% for 10 μM and 70.9% for 100 μM concentrations.

### 3.3 AP‐1 Is Required for Mmps Expression

As discussed above, *mmps* expression appears between 3–6 h post injury (intermediate phase). However, an earlier wave of gene expression occurs around 1 h. This earlier wave includes genes required for metabolic changes and stress response. Stress is usually accompanied by the expression of AP‐1 components such as *jun* and *fos* genes. Previous literature has already suggested that there is a connection between AP‐1 and *mmps*, and our goal was to determine whether it is also the case during embryonic healing.

**FIGURE 4:**
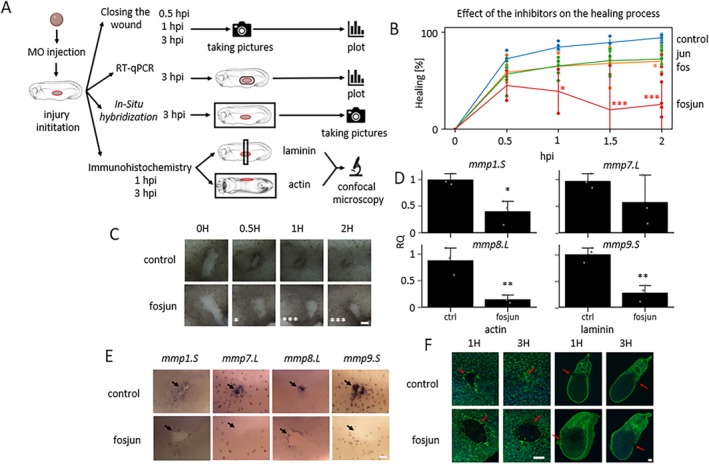
Importance of AP‐1 during embryonic wound healing. (A) Scheme of the experimental procedure. (B) Quantification of wound closure in AP‐1 loss of function embryos in time (x axis), calculated as percentage normalised to time 0 (*n* = 2–14), two‐way ANOVA followed by Dunnet's post hoc test, *p* < 0.05 *, *p* < 0.01 **, *p* < 0.001 ***. (C) Brightfield images of wound assays in AP‐1 loss of function. Showed are representative embryos (*n* = 10, scale bar 100 μm). (D) The effect of AP‐1 loss of function on *mmps* expression studied by RT‐qPCR at 3 h after the injury (*n* = 3), one‐way ANOVA followed by Dunnet's post hoc test, *p* < 0.05 *, *p* < 0.01 **, p < 0.001 ***. (E) Validation of *mmps* expression using in situ hybridisation (*n* = 10, scale bar 50 μm). (F) Actin ring formation defect (phalloidin staining) and the problem of basal lamina formation showed with the red arrow (Laminin staining) (*n* = 10, scale bar 100 μm). (fosjun—Morpholino targeted the combination of *fos* and *jun* genes).

We used MOs to inhibit two well‐known AP‐1 genes: *fos* and *jun*. We know from bulk RNA‐Seq that both are highly expressed during the early healing phase [10]. We targeted, through injections, a single gene (L/S) or a combination of different genes, followed by wound assay, and found that it decreased the healing capacity. Individual injection of *fos* or *jun* MO led to wound closure at the level of 70% and 72.3%, respectively (Figure 4B). However, a combination of *fos*: *jun* MOs at a concentration of 3:1 showed a much higher effect of reducing the ability of closure (25.6%) (Figure 4B,C). We performed an RT‐qPCR experiment to quantify the level of *mmps* expression dependence on AP‐1 activity. AP‐1 loss of function embryos showed significantly decreased expression of *mmp1.S*, *mmp8.L* and *mmp9.S*, while no significant decrease for *mmp7.L* (Figure 4D). We validated these results by in situ hybridisation and found reduction of staining at the wound site for *mmp1* and *mmp8* and myeloid cell defect using *mmp7* and *mmp9* staining (Figure 4E). In addition, AP‐1 loss of function embryos showed also defects in actin ring formation and Laminin layer formation (Figure 4F).

### 3.4 Expression of Mmp Genes Showed Similarities Among Models

**FIGURE 5:**
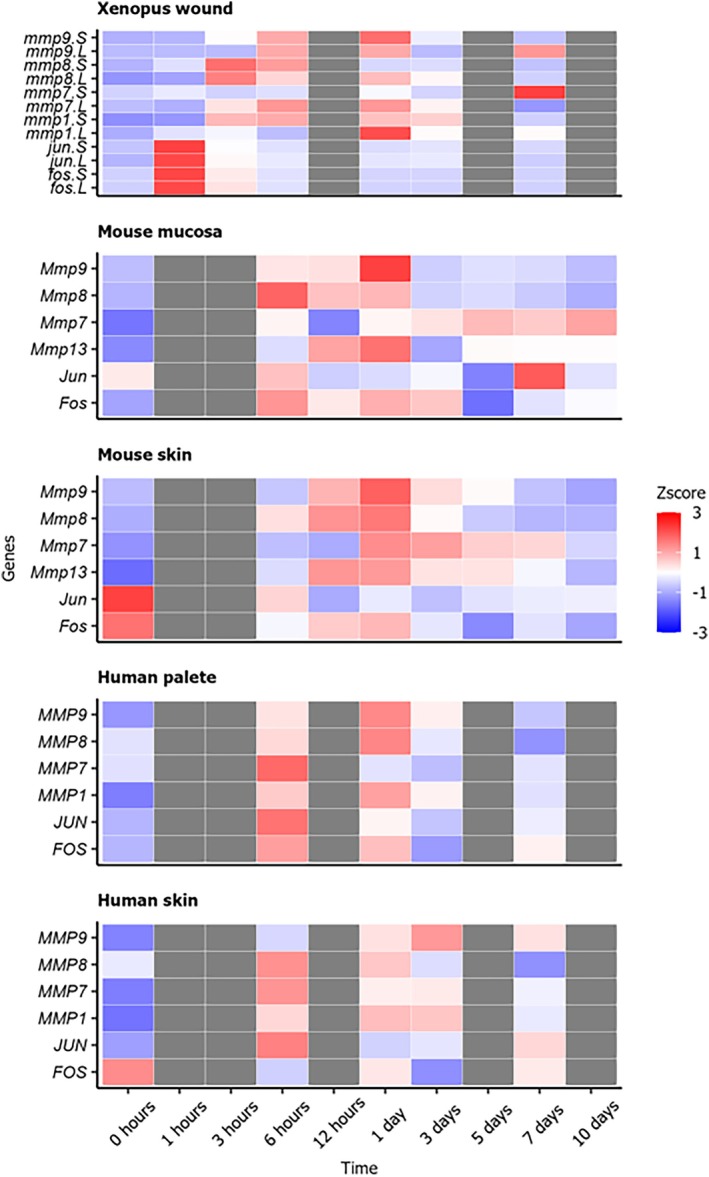
Comparison of *mmps* and AP‐1 genes expression studied from microarray datasets. *Xenopus*, mouse and human datasets were reanalysed and showed similarities during early phase (AP‐1 signalling) and intermediate phase (*mmps*). In the mouse, *Mmp13* serves as an equivalent to *Mmp1*.

To study the conservation of wound healing regulation between our *Xenopus* model and mammalian healing datasets, we utilised available microarray studies from mouse [26] and human studies [25]. Expression of AP‐1 genes (*fos* and *jun*) showed similar rapid increase during early healing phase and then decrease in later phases of healing. Surprisingly, the maximum level of both genes was observed at time 0 in mouse and human skin samples compared to 1 h post injury in *Xenopus*. The same peak at 1 h post injury was found in mouse mucosa and human palate samples. Good correlation was observed among *mmp* genes during intermediate phase of healing. They showed peak expression between 3 h and 1 day post injury followed by decrease of expression in the later phases in all studied tissues and models (Figure 5).

### 3.5 Gene Expression of Mmps and AP‐1 Genes Showed Similarity Also at Cellular Level

**FIGURE 6:**
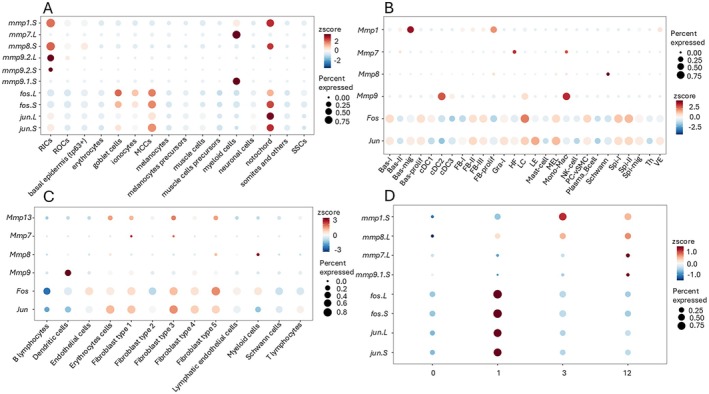
Expression of AP‐1 and *mmp* genes studied at single cell level. (A) Single cell RNA‐seq from *Xenopus* healing (0–12 h post injury) showed expression of *mmps* in epidermal and myeloid cells and broader expression of *fos*/*jun*. (RIC—regeneration initiating cells, ROC—regeneration organising cells, MCCs—multicilliated cells, SSCs—secretory cells). (B) Human single cell study (intact skin, 1 day, 7 days and 30 days post injury) of wound healing showed broad expression of AP‐1 and cell specific expression of *Mmps*. (DC‐ dendritic cells, HF—heart failure cells, LC—langerhans cells, LE—lupus erythematosus cells). (C) Mouse single cell study (12 days post injury) of wound healing showed broad expression of AP‐1 and cell specific expression of *Mmps*. (D) Temporal average expression of genes of interest from our *Xenopus* single cell regeneration study (time 0, 1, 3 and 12 h).

We compared the average expression of the genes of interest at the individual cell cluster level to reveal differences and similarities between *Xenopus* and mammalian healing models. Recently, we published a single cell analysis of regeneration in *Xenopus*, where we focused on the early regeneration phase, which in many aspects relates to wound healing [27]. In *Xenopus*, we found *mmp1.S* and *mmp8.L* to be expressed in a sub‐group of epidermal cells (called RICs) and notochord cells, while *mmp7.L* and *mmp9.S* to be expressed in myeloid cells. Genes coding *fos* and *jun* showed broader expression with primary expression in skin cells (goblet, ionocytes and multicilliated cells) and notochord (Figure 6A). Human single cell analysis of wound healing [29] showed broad expression of *fos* and *jun* and cell‐specific expression of *Mmps* (*Mmp1* in skin (keratinocyte) population); expression of *Mmp7* and *Mmp9* in myeloid cells (dendritic, monocytes and macrophage cells) (Figure 6B). Mouse single cell analysis of wound healing [29] showed again expression of *fos*/*jun* in many cell types, in contrast to specific expression of *Mmps*. *Mmp13*, which is supposed to substitute *Mmp1* in mouse [28, 29] and *Mmp7* were expressed in fibroblasts, *Mmp8* and *Mmp9* were predominant in myeloid and dendritic cells, respectively (Figure 6C).

We also checked the average expression of the genes of interest across the analysed time points from our *Xenopus* single cell data to confirm our observation of injury‐induced increase in expression of *mmp1* and *mmp8* in contrast to stable expression of *mmp7* and *mmp9* (Figure 6D). Both AP‐1 genes showed increased expression at 1 h post injury, *mmp1* and *mmp8* showed increase of expression post injury at 3 and 12 h. In contrast, *mmp7* and *mmp9* expression remained stable during 1 and 3 h, and an increase could be seen at 12 h, corresponding to the potential of myeloid cell migration to the wound area.

## 4 Discussion

Wound healing is an interesting biological process. Although the embryonic type appears simple, it is complex and involves many phenotypic changes in surrounding cells and broad gene expression activity. Several waves of new gene expression can be observed, with the first early wave occurring 30 to 60 min after injury. This initial wave is characterised by stress response genes [33], including the AP1 pathway, mainly composed of *jun* and *fos* genes. These early response genes are essential for cell migration and re‐epithelialisation [34, 35] and help promote the expression of additional genes with AP‐1 binding sites, such as keratins and matrix metalloproteinases [36, 37, 38, 39]. The role of AP1 has also been studied during later developmental stages of *Xenopus* and was found to be important for healing and regeneration [40, 41]. The early gene expression wave is supported by actin‐myosin ring formation and rapid wound constriction.

The second wave of gene expression occurs 3 to 6 h after injury [10] and is characterised by a defence response, activation of cellular processes such as proliferation or cell death, and remodelling of the ECM. MMPs are likely key enzymes involved in ECM remodelling in *Xenopus* embryos. This is also observed in *Danio rerio* , which also uses *mmp9* expression to change the ECM. *mmp9* may also influence cytokine signalling during healing [42].

Based on our *Xenopus* bulk RNA‐Seq data, at least 15 *mmp* genes are involved in wound healing. We selected the four most prominent *mmps*: *mmp1, mmp7, mmp8* and *mmp9* for further analysis. We observed an increase of these *mmps* around the 3 h post injury at the site of the wound. Similarly, in mouse and human healing datasets, these *mmps* also showed an increase in their expression at the wound site following injury. In *Xenopus* embryos, *mmp7* and *mmp9* are expressed in myeloid cells, which migrate to the wound area [43]. In contrast to myeloid cell migration and accumulation of *mmp7* and *mmp9* after injury, we found that in *Xenopus* embryonic healing, the *mmp1* and *mmp8* are expressed in epithelial cells around the wound area. We also observed that *mmp* genes are responsible for closing the wound and remodelling the Laminin layer.

The cross‐species conservation of these *mmps* expression after injury may be explained by their functional roles. The role of Mmp1 during wound healing was shown in mammals to accelerate epithelisation, reduce inflammation and increase vascularisation. It also reduces the scar formation [44, 45]. The expression of *Mmp1* in the basal keratinocytes in the front part of the wound is triggered by the loss of ECM [46]. It was also recently suggested to be substituted by the activity of *Mmp13* in mouse [29]. A strong increase in Mmp8 has also been documented in other healing models [47], and it has been indicated that *Mmp8* contributes to inflammation [48]. It has been shown that Mmp7 in mammals is involved in re‐epithelialisation. It also cleaves ECM or ECM‐associated proteins and facilitates keratinocyte migration. Mmp9 is in mammal wound healing crucial for collagen reorganisation and keratinocyte migration [12].

Recent single cell studies in mammals also showed differences among expression of *mmps* during healing. Surprisingly, human wound healing reflects a similar wound‐healing process as is in *Xenopus*, but mouse healing differs. For example, myeloid (immune) cells express *mmp7/Mmp7* and *mmp9*/*Mmp9* in both human and *Xenopus*, but mouse study showed rather the expression of a combination of *Mmp8* and *Mmp9* in immune cells.

Axolotl has more *mmp* genes than is typical for most vertebrates (including humans). Some *mmp* genes are exclusive for the salamander lineage only [49]. As well as *Xenopus*, they both have more *mmp* genes, which may lead to better healing ability. The expression levels of some *mmps*, such as *mmp2* and *mmp9*, persist long after injury and support their important role in matrix remodelling [50]. Other studies focusing on the importance of the healing process showed the involvement of additional *mmps* which were not enriched in *Xenopus* embryos. The expression of mouse *Mmp3* was expressed in proliferating cells adjacent to the wound edge and was described as important for wound contraction [51]. Also, the expression of *Mmp10* was found to colocalise with *Mmp1* in healing mammalian epidermis. A decrease of *Mmp10* leads to a disorganised migrating epithelium, degradation of newly formed matrix, aberrant cell–cell contacts of the migrating keratinocytes, and an increased rate of cell death of wound edge keratinocytes [52]. We assume that the wide range of remodelling enzymes with likely species‐specific activation will lead to significant challenges in studies trying to understand the individual healing mechanisms.

**FIGURE 7:**
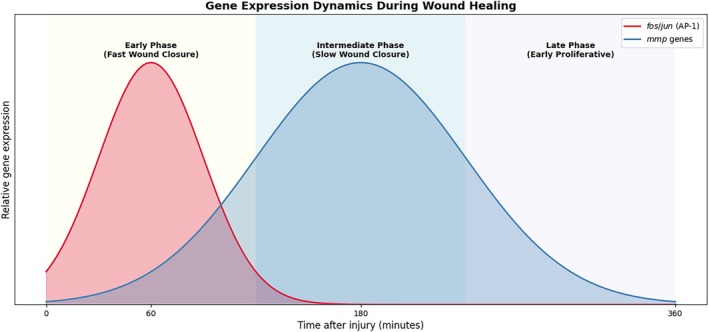
Summary of AP1 and *mmps* roles during embryonic wound healing.

In summary, our study showed that the combination and dependence of AP‐1 and *mmps* expression are crucial for embryonic wound healing. Based on recent single cell studies in human [29] and *Xenopus* [27], we can also speculate about more complex regulation of AP‐1 and downstream *mmps*. Study of healing in *Xenopus* revealed potential regulation of wound specific *mmps* and ECM components controlled by cell co‐expression of *junb* and *fosl1*. In human healing study, authors found similar master regulator FOSL1 to be required for efficient wound healing. Taken together, AP‐1 and *mmps* are responsible for initiating wound closure and tissue remodelling (Figure 7). The disruption of *mmps* expression causes the incompetence of myeloid cells, which migrate to the wound area and prevent the formation of the actin‐myosin ring in the edge epithelial cells, resulting in a wound closure defect.

## 5 Conclusion

We were able to provide the first detailed temporal analysis of specific *mmp* genes (*mmp1.S, mmp7.L, mmp8.L* and *mmp9.S*) in wound healing in *Xenopus laevis* embryos showing their importance. We also demonstrated that *mmp* genes functionally depend on the early AP‐1 activation pathway. These results support the connection of early stress responses directly to ECM remodelling enzymes. Our integration of bulk RNA‐seq and single cell RNA‐seq confirms that *mmps* expression patterns observed in *Xenopus laevis* are conserved at the cellular level also in mammalian wound healing. This shows potential in therapeutic targeting.

## Author Contributions

Daniel Kraus: conceptualisation, methodology, investigation, validation, formal analysis, data curation, data analysis, writing – original draft, visualisation; Paulina Kikinderova: investigation; Pavel Abaffy: investigation, analysis, writing; Dominika Kadlcikova: investigation; Ravindra Naraine: data analysis, writing; Radek Sindelka: conceptualisation, methodology, validation, writing – original draft, visualisation, supervision, funding acquisition.

## Funding

This work was supported by Grantová Agentura České Republiky (24‐12027S), Ministerstvo Zdravotnictví Ceské Republiky (NW24‐03‐00459), Imaging Methods Core Facility at BIOCEV (LM2018129, MEYS CR), RVO (86652036), MULTIOMICS_CZ (Programme Johannes Amos Comenius), MEYS CR – The European Union, (CZ.02.01.01/00/23_020/0008540).

## Conflicts of Interest

The authors declare no conflicts of interest.

## Supporting information


**Figure S1:** Expression of mmp genes in different cell types. Single cell RNA‐seq data show which mmp genes are expressed in which cell type

## Data Availability

The data that support the findings of this study are available on request from the corresponding author. The data are not publicly available due to privacy or ethical restrictions.

## References

[1] P. Martin , “Wound Healing–Aiming for Perfect Skin Regeneration,” Science 276, no. 5309 (1997): 75–81. 9082989 10.1126/science.276.5309.75

[2] B. J. Larson , M. T. Longaker , and H. P. Lorenz , “Scarless Fetal Wound Healing: A Basic Science Review,” Plastic and Reconstructive Surgery 126, no. 4 (2010): 1172–1180, 10.1097/PRS.0b013e3181eae781. 20885241 PMC4229131

[3] S. Bunman , N. Dumavibhat , W. Chatthanawaree , S. Intalapaporn , T. Thuwachaosuan , and C. Thongchuan , “Burn Wound Healing: Pathophysiology and Current Managementof Burn Injury,” Bangkok Medical Journal 13, no. 2 (2017): 91.

[4] W. G. Owen , C. T. Esmon , and C. M. Jackson , “THE Conversion of Prothrombin to Thrombin: I. CHARACTERIZATION OF THE REACTION PRODUCTS FORMED DURING THE ACTIVATION OF BOVINE PROTHROMBIN,” Journal of Biological Chemistry 249, no. 2 (1974): 594–605. 4809530

[5] D. M. Monroe and M. Hoffman , “What Does It Take to Make the Perfect Clot?,” Arteriosclerosis, Thrombosis, and Vascular Biology 26, no. 1 (2006): 41–48. 16254201 10.1161/01.ATV.0000193624.28251.83

[6] G. S. Schultz , G. A. Chin , L. Moldawer , and R. F. Diegelmann , “Principles of Wound Healing,” in Mechanisms of Vascular Disease: A Reference Book for Vascular Specialists [Internet] (University of Adelaide Press, 2011). 30485016

[7] P. Cowled and R. Fitridge , “Pathophysiology of Reperfusion Injury,” in Mechanisms of Vascular Disease: A Reference Book for Vascular Specialists [Internet] (University of Adelaide Press, 2011). 30484990

[8] N. X. Landén , D. Li , and M. Ståhle , “Transition From Inflammation to Proliferation: A Critical Step During Wound Healing,” Cellular and Molecular Life Sciences 73 (2016): 3861–3885. 27180275 10.1007/s00018-016-2268-0 PMC5021733

[9] A. Leung , T. M. Crombleholme , and S. G. Keswani , “Fetal Wound Healing: Implications for Minimal Scar Formation,” Current Opinion in Pediatrics 24, no. 3 (2012): 371–378, 10.1097/MOP.0b013e3283535790. 22572760 PMC4528185

[10] P. Abaffy , S. Tomankova , R. Naraine , M. Kubista , and R. Sindelka , “The Role of Nitric Oxide During Embryonic Wound Healing,” BMC Genomics 20, no. 1 (2019): 815. 31694542 10.1186/s12864-019-6147-6 PMC6836512

[11] H. Weavers , W. Wood , and P. Martin , “Injury Activates a Dynamic Cytoprotective Network to Confer Stress Resilience and Drive Repair,” Current Biology 29, no. 22 (2019): 3851–3862.e4. 31668626 10.1016/j.cub.2019.09.035 PMC6868510

[12] M. P. Caley , V. L. C. Martins , and E. A. O'Toole , “Metalloproteinases and Wound Healing,” Advances in Wound Care 4, no. 4 (2015): 225–234. 25945285 10.1089/wound.2014.0581 PMC4397992

[13] J. M. P. Freije , M. Balbín , A. M. Pendás , et al., “Matrix Metalloproteinases and Tumor Progression,” in New Trends in Cancer for the 21st Century: Proceedings of the International Symposium on Cancer: New Trends in Cancer for the 21st Century, Held November 10–13, 2002, in Valencia, Spain [Internet], ed. A. Llombart‐Bosch and V. Felipo (Springer US, 2003), 91–107. Available from, 10.1007/978-1-4615-0081-0_9.

[14] J. Hu , P. E. den Van Steen , Q. X. A. Sang , and G. Opdenakker , “Matrix Metalloproteinase Inhibitors as Therapy for Inflammatory and Vascular Diseases,” Nature Reviews Drug Discovery 6, no. 6 (2007): 480–498. 17541420 10.1038/nrd2308

[15] D. Q. Li , B. L. Lokeshwar , A. Solomon , D. Monroy , Z. Ji , and S. C. Pflugfelder , “Regulation of MMP‐9 Production by Human Corneal Epithelial Cells,” Experimental Eye Research 73, no. 4 (2001): 449–459. 11825017 10.1006/exer.2001.1054

[16] C. M. Overall and C. López‐Otín , “Strategies for MMP Inhibition in Cancer: Innovations for the Post‐Trial Era,” Nature Reviews. Cancer 2, no. 9 (2002): 657–672. 12209155 10.1038/nrc884

[17] U. Benbow and C. E. Brinckerhoff , “The AP‐1 Site and MMP Gene Regulation: What Is All the Fuss About?,” Matrix Biology 15, no. 8 (1997): 519–526. 9138284 10.1016/s0945-053x(97)90026-3

[18] T. M. Hess , J. T. Hinson , and J. A. Statham , “Explicit and Implicit Stereotype Activation Effects on Memory: Do Age and Awareness Moderate the Impact of Priming?,” Psychology and Aging 19, no. 3 (2004): 495–505. 15382999 10.1037/0882-7974.19.3.495

[19] M. Ameyar , M. Wisniewska , and J. B. Weitzman , “A Role for AP‐1 in Apoptosis: The Case for and Against,” Biochimie 85, no. 8 (2003): 747–752. 14585541 10.1016/j.biochi.2003.09.006

[20] E. F. Wagner , “AP‐1 – Introductory Remarks,” Oncogene 20, no. 19 (2001): 2334–2335. 11402330 10.1038/sj.onc.1204416

[21] R. Eferl and E. F. Wagner , “AP‐1: a double‐edged sword in tumorigenesis,” Nature Reviews. Cancer 3, no. 11 (2003): 859–868. 14668816 10.1038/nrc1209

[22] E. K. O'Shea , R. Rutkowski , and P. S. Kim , “Mechanism of Specificity in the Fos‐Jun Oncoprotein Heterodimer,” Cell 68, no. 4 (1992): 699–708. 1739975 10.1016/0092-8674(92)90145-3

[23] B. Grondin , D. G. T. M. Lefrancois , M. Tremblay , et al., “C‐Jun Homodimers Can Function as a Context‐Specific Coactivator,” Molecular and Cellular Biology 27, no. 8 (2007): 2919–2933. 17283046 10.1128/MCB.00936-06 PMC1899927

[24] H. L. Sive , R. M. Grainger , and R. M. Harland , Early Development of Xenopus Laevis : A Laboratory Manual (Cold Spring Harbor Laboratory Press, 2000), https://api.semanticscholar.org/CorpusID:84634958.

[25] T. R. Leonardo , L. Chen , M. E. Schrementi , et al., “Transcriptional Changes in Human Palate and Skin Healing,” Wound Repair and Regeneration 31, no. 2 (2023): 156–170. 36571451 10.1111/wrr.13068 PMC10006330

[26] L. Chen , Z. H. Arbieva , S. Guo , P. T. Marucha , T. A. Mustoe , and L. A. DiPietro , “Positional Differences in the Wound Transcriptome of Skin and Oral Mucosa,” BMC Genomics 11, no. 1 (2010): 471. 20704739 10.1186/1471-2164-11-471 PMC3091667

[27] R. Sindelka , R. Naraine , P. Abaffy , et al., “Characterization of Regeneration Initiating Cells During *Xenopus laevis* Tail Regeneration,” Genome Biology 25, no. 1 (2024): 251. 39350302 10.1186/s13059-024-03396-3 PMC11443866

[28] C. F. Guerrero‐Juarez , P. H. Dedhia , S. Jin , et al., “Single‐Cell Analysis Reveals Fibroblast Heterogeneity and Myeloid‐Derived Adipocyte Progenitors in Murine Skin Wounds,” Nature Communications 10, no. 1 (2019): 650. 10.1038/s41467-018-08247-x PMC6368572 30737373

[29] Z. Liu , X. Bian , L. Luo , et al., “Spatiotemporal Single‐Cell Roadmap of Human Skin Wound Healing,” Cell Stem Cell 32, no. 3 (2025): 479–498.e8. 39729995 10.1016/j.stem.2024.11.013

[30] B. C. Kieseier , J. M. Clements , H. B. Pischel , et al., “Matrix Metalloproteinases MMP‐9 and MMP‐7 Are Expressed in Experimental Autoimmune Neuritis and the Guillain‐barré Syndrome,” Annals of Neurology 43, no. 4 (1998): 427–434. 9546322 10.1002/ana.410430404

[31] J. Chou , M. F. Chan , and Z. Werb , “Metalloproteinases: A Functional Pathway for Myeloid Cells,” Microbiology Spectrum 4, no. 2 (2016), 10.1128/microbiolspec.MCHD-0002-2015. PMC4888795 27227311

[32] M. Kandhwal , T. Behl , S. Singh , et al., “Role of Matrix Metalloproteinase in Wound Healing,” American Journal of Translational Research 14, no. 7 (2022): 4391–4405. 35958464 PMC9360851

[33] A. Thieffry , D. López‐Márquez , J. Bornholdt , et al., “PAMP‐Triggered Genetic Reprogramming Involves Widespread Alternative Transcription Initiation and an Immediate Transcription Factor Wave,” Plant Cell 34, no. 7 (2022): 2615–2637. 35404429 10.1093/plcell/koac108 PMC9252474

[34] P. Jaakkola , S. Kontusaari , T. Kauppi , A. Määttä , and M. Jalkanen , “Wound Reepithelialization Activates a Growth Factor‐Responsive Enhancer in Migrating Keratinocytes,” FASEB Journal 12, no. 11 (1998): 959–969. 9707168 10.1096/fasebj.12.11.959

[35] P. O. T. Tran , L. E. Hinman , G. M. Unger , and P. J. Sammak , “A Wound‐Induced [Ca2+]iIncrease and Its Transcriptional Activation of Immediate Early Genes Is Important in the Regulation of Motility,” Experimental Cell Research 246, no. 2 (1999): 319–326. 9925747 10.1006/excr.1998.4239

[36] P. Angel , A. Szabowski , and M. Schorpp‐Kistner , “Function and Regulation of AP‐1 Subunits in Skin Physiology and Pathology,” Oncogene 20, no. 19 (2001): 2413–2423. 11402337 10.1038/sj.onc.1204380

[37] S. Yates and T. E. Rayner , “Transcription Factor Activation in Response to Cutaneous Injury: Role of AP‐1 in Reepithelialization,” Wound Repair and Regeneration 10, no. 1 (2002): 5–15. 11983002 10.1046/j.1524-475x.2002.10902.x

[38] L. Florin , L. Hummerich , B. T. Dittrich , et al., “Identification of Novel AP‐1 Target Genes in Fibroblasts Regulated During Cutaneous Wound Healing,” Oncogene 23, no. 42 (2004): 7005–7017. 15273721 10.1038/sj.onc.1207938

[39] L. Florin , J. Knebel , P. Zigrino , et al., “Delayed Wound Healing and Epidermal Hyperproliferation in Mice Lacking JunB in the Skin,” Journal of Investigative Dermatology 126, no. 4 (2006): 902–911. 16439969 10.1038/sj.jid.5700123

[40] M. Nakamura , H. Yoshida , E. Takahashi , et al., “The AP‐1 Transcription Factor JunB Functions in Xenopus Tail Regeneration by Positively Regulating Cell Proliferation,” Biochemical and Biophysical Research Communications 522, no. 4 (2020): 990–995. 31812242 10.1016/j.bbrc.2019.11.060 PMC6989358

[41] T. Tamaki , T. Yoshida , E. Shibata , H. Nishihara , H. Ochi , and A. Kawakami , “Splashed E‐Box and AP‐1 Motifs Cooperatively Drive Regeneration Response and Shape Regeneration Abilities,” Biology Open 12, no. 2 (2023): bio059810. 36636913 10.1242/bio.059810 PMC9922731

[42] N. J. Silva , M. Nagashima , J. Li , et al., “Inflammation and Matrix Metalloproteinase 9 (Mmp‐9) Regulate Photoreceptor Regeneration in Adult Zebrafish,” Glia 68 (2020): 1445–1465. 32034934 10.1002/glia.23792 PMC7317489

[43] R. M. B. Costa , X. Soto , Y. Chen , A. M. Zorn , and E. Amaya , “Spib Is Required for Primitive Myeloid Development in Xenopus,” Blood 112, no. 6 (2008): 2287–2296. 18594023 10.1182/blood-2008-04-150268 PMC2577559

[44] E. S. Keskin , E. R. Keskin , M. B. Öztürk , and D. Çakan , “The Effect of MMP‐1 on Wound Healing and Scar Formation,” Aesthetic Plastic Surgery 45, no. 6 (2021): 2973–2979. 34075460 10.1007/s00266-021-02369-2

[45] B. K. Pilcher , J. A. Dumin , B. D. Sudbeck , S. M. Krane , H. G. Welgus , and W. C. Parks , “The Activity of Collagenase‐1 Is Required for Keratinocyte Migration on a Type I Collagen Matrix,” Journal of Cell Biology 137, no. 6 (1997): 1445–1457. 9182674 10.1083/jcb.137.6.1445 PMC2132537

[46] Y. Iimuro , T. Nishio , T. Morimoto , et al., “Delivery of Matrix Metalloproteinase‐1 Attenuates Established Liver Fibrosis in the Rat,” Gastroenterology 124, no. 2 (2003): 445–458. 12557150 10.1053/gast.2003.50063

[47] B. Hartenstein , B. T. Dittrich , D. Stickens , et al., “Epidermal Development and Wound Healing in Matrix Metalloproteinase 13‐Deficient Mice,” Journal of Investigative Dermatology 126, no. 2 (2006): 486–496. 16374453 10.1038/sj.jid.5700084 PMC2767339

[48] A. Gutiérrez‐Fernández , M. Inada , M. Balbín , et al., “Increased Inflammation Delays Wound Healing in Mice Deficient in Collagenase‐2 (MMP‐8),” FASEB Journal 21, no. 10 (2007): 2580–2591. 17392479 10.1096/fj.06-7860com PMC2575772

[49] N. Al Haj Baddar , N. Timoshevskaya , J. J. Smith , H. Guo , and S. R. Voss , “Novel Expansion of Matrix Metalloproteases in the Laboratory Axolotl (*Ambystoma mexicanum*) and Other Salamander Species,” Frontiers in Ecology and Evolution [Internet] 9. Available from (2021), https://www.frontiersin.org/journals/ecology‐and‐evolution/articles/10.3389/fevo.2021.786263.

[50] S. Arumugam , Y. C. Jang , C. Chen‐Jensen , N. S. Gibran , and F. F. Isik , “Temporal Activity of Plasminogen Activators and Matrix Metalloproteinases During Cutaneous Wound Repair,” Surgery 125, no. 6 (1999): 587–593. 10372023

[51] K. Bullard , L. Lund , J. Mudgett , et al., “Impaired Wound Contraction in Stromelysin‐1‐Deficient Mice,” Annals of Surgery 230, no. 2 (1999): 260–265. 10450741 10.1097/00000658-199908000-00017 PMC1420869

[52] M. Krampert , W. Bloch , T. Sasaki , et al., “Activities of the Matrix Metalloproteinase Stromelysin‐2 (MMP‐10) in Matrix Degradation and Keratinocyte Organization in Wounded Skin,” Molecular Biology of the Cell 15, no. 12 (2004): 5242–5254. 15371548 10.1091/mbc.E04-02-0109 PMC532007

