# Supplementary material for: Regulation of Embryonic Wound Healing by Matrix Metalloproteinases in Xenopus laevis Tailbud Stage
Source: Wound Repair Regen. 2026 Feb 18;34(1):e70134. doi: 10.1111/wrr.70134 (PMC12917100; doi:10.1111/wrr.70134)
Supplement: Supplementary file 1 — Figure S1: Expression of mmp genes in different cell types. Single cell RNA‐seq data show which mmp genes are expressed in which cell type [file WRR-34-0-s001.pdf]

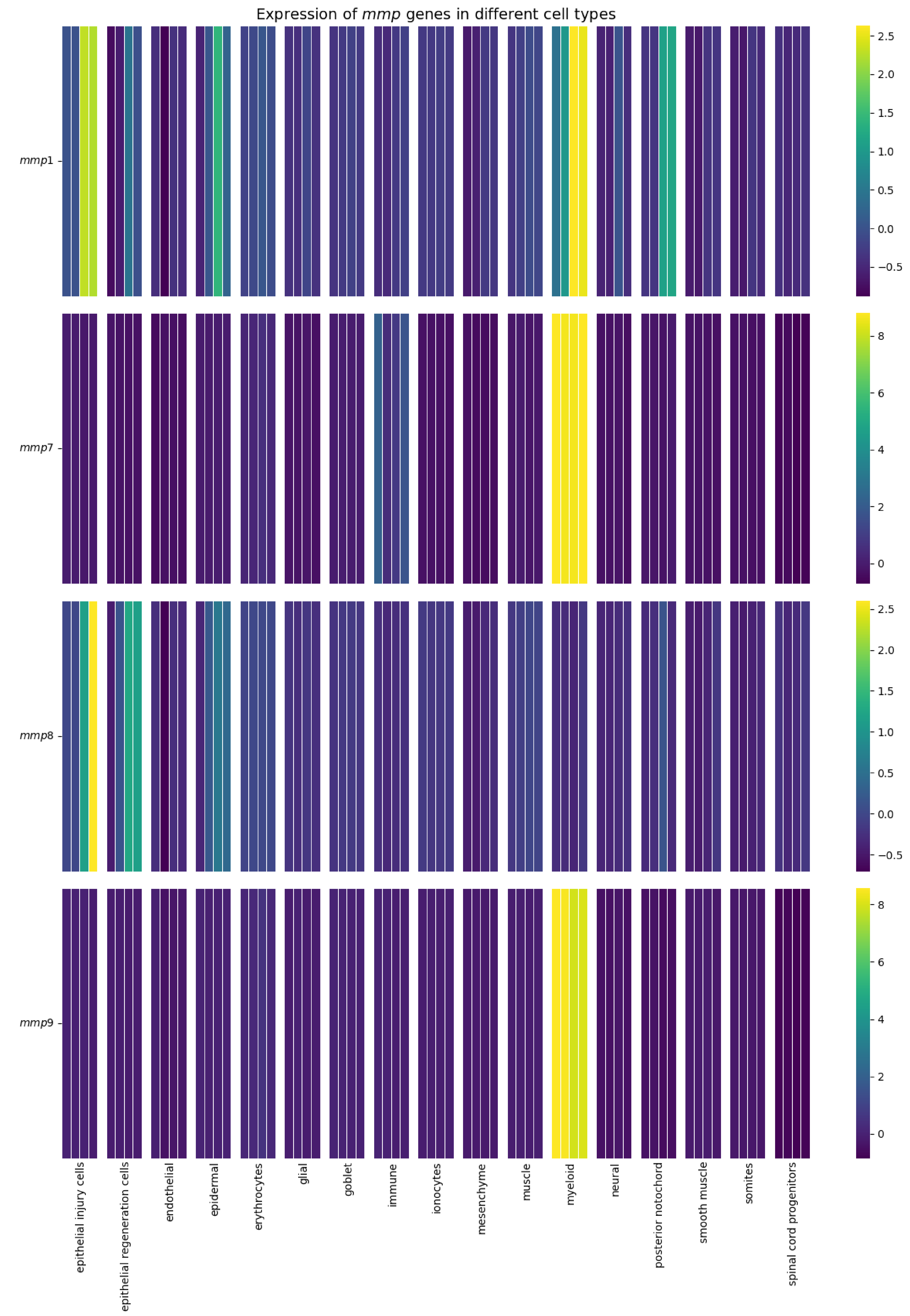

Supplementary Figure 1. Expression of *mmp* genes in different cell types. Single cell RNA-seq data show which *mmp* genes are expressed in which cell type
